# Stacking Order-Dependent Electronic and Optical Properties of h-BP/Borophosphene Van Der Waals Heterostructures

**DOI:** 10.3390/nano15151155

**Published:** 2025-07-25

**Authors:** Kejing Ren, Quan Zhang, Shengli Zhang, Yang Zhang

**Affiliations:** Ministry of Education Key Laboratory for Nonequilibrium Synthesis and Modulation of Condensed Matter, School of Physics, Xi’an Jiaotong University, Xi’an 710049, Chinazhangsl@xjtu.edu.cn (S.Z.)

**Keywords:** vdW heterostructure, structural stability, electronic property, optical absorption spectra, first-principles study

## Abstract

Van der Waals (vdW) heterostructures, typically composed of two-dimensional (2D) atomic layers, have attracted significant attention over the past few decades. Their performance is closely dependent on their composition and interlayer interactions. In this study, we constructed four types of 2D hexagonal BP monolayer (h-BP)/borophosphene vdW heterostructures with different stacking orders: (i) B-B stacking, (ii) P-P stacking, (iii) moire-I, and (iv) moire-II. Their structural stability and their electronic and optical properties were explored by using first-principles calculations. The results show that h-BP/borophosphene heterostructures can maintain their configurations with good structural stability and minimal lattice mismatch. All vdW heterostructures exhibit semiconducting characteristics, and their band gaps are highly dependent on interlayer stacking orders. Due to the regular atomic arrangement and enhanced interlayer dipole interactions, the B-B stacking bilayer opens a relatively large band gap of 0.157 eV, while the moire-II bilayer exhibits a very small band gap of 0.045 eV because of its irregular atom arrangements. By calculating the complex dielectric function, optical absorption spectra of B-B and P-P stacking bilayers were discussed. This study suggests that h-BP/borophosphene heterostructures have desirable optical properties, broadening the potential applications of the constituent monolayers.

## 1. Introduction

Since the experimental synthesis of single-layered graphene in 2004, research into two-dimensional (2D) nanomaterials has grown in the past few decades [1]. Various 2D nanomaterials, such as silicene [2], germanene [3], carbon allotropes [4,5], transition metal disulfide compounds [6], hexagonal boron nitride [7], and so on, have been proposed and synthesized experimentally. Due to their unique electronic, optical, and magnetic properties, some of them show great potential applications in electronic devices, flexible electronics, sensors, and energy storage systems [8,9]. However, in terms of achieving high-performance devices, the properties of a single 2D nanomaterial are hardly satisfactory. Heterostructures are promising candidates. They can not only integrate the advantages of various 2D materials to compensate for their disadvantages but they can also generate other unique physical properties and phenomena to expand their potential applications [10,11].

Van der Waals (vdW) heterostructures are typically composed of 2D layered materials stacked in a precisely selected sequence, and their performance closely depends on their constituents and the interlayers’ physical interactions. Weak interlayer interactions have enabled the successful use of mechanical exfoliation and transfer technologies to produce vdW heterostructures, making this field increasingly popular in recent research [12]. In 2014, Hong et al. observed ultrafast charge transfer in photoexcited MoS_2_/WS_2_ vdW heterostructures for the first time [13]. They used photoluminescence mapping and femtosecond pump-probe spectroscopy. This rapid charge transfer, occurring within a femtosecond, could lead to innovative 2D devices for optoelectronics and light harvesting based on vdW heterostructures. By forming a vdW heterostructure with graphene, the excitation transfer processes in WS_2_ can be greatly improved [14], which is essential for applications in photovoltaics and photodetectors. In addition, many graphene-based heterostructures have been proposed theoretically, such as borophene/Graphene [15], borophosphene/graphene [16,17], phosphorene/Graphene [18], graphene/hexagonal III-V monolayer (GaP, GaAs, InP, and InAs) [19], and so on. However, all the vdW heterostructures mentioned above possess a basic characteristic, which is that they are constructed from different layered materials composed of different elements. Few works are devoted to exploring the vdW heterostructures formed between allotropies, such as 1T’/2H-MoS_2_ vdW heterostructures [20], which allow for the possibility of forming a coherent interface with no lattice mismatch.

Recently, a new 2D Dirac monolayer of borophosphene has been proposed. It has been suggested to be stable mechanically, thermally, and dynamically and is composed of light elements of B and P [21]. Interestingly, the Dirac cone of borophosphene is robust and independent of in-plane biaxial and uniaxial strains, exhibiting high electronic conductivity with a Fermi velocity of 10^5^ m/s. Another allotropy composed of B and P elements is a graphene-like hexagonal honeycomb monolayer (h-BP), which has been predicted to exfoliate from the BP (111) surface [22]. The h-BP monolayer exhibits a moderate direct band gap of 1.37 eV [23], while borophosphene is a semimetal [21]. Despite their differing geometric symmetries, the structural parameters of h-BP and borophosphene exhibit significant similarities, suggesting a substantial potential for the formation of vdW heterostructures with minimal lattice mismatch. The stacking order, including superlattice arrangement, has been demonstrated to be an effective method for modulating interface coupling [24,25,26]. Moreover, the optical absorption can be enhanced in VdW heterostructures [27,28]. However, vdW heterostructures composed of isomeric nanostructures (e.g., h-BP and borophosphene) have been seldom studied. It is anticipated that multiple types of stacking orders, or interface coupling, may exist in these heterostructures.

In this study, we will employ first-principles calculations to explore the structural stability and the electronic and optical properties of 2D h-BP/borophosphene vdW heterostructures. Four types of heterostructures with different stacking orders or superlattice arrangements are considered, showing good structural stability and minimal lattice mismatches. Since the interlayer interaction alters the π and π* bonding characteristics of borophosphene, all vdW heterostructures exhibit narrow band gap semiconducting. Their band gaps closely depend on the stacking order. In addition, anisotropic and enhanced optical properties are observed in the h-BP/borophosphene vdW heterostructures.

## 2. Computational Methods

All calculations were performed by using first-principles study based on the spin-polarized density functional theory (DFT) within the projector augmented wave method [29,30], as implemented in the Vienna ab initio simulation package (VASP) [31,32]. The generalized gradient approximation (GGA) with the functional of Perdew–Burke–Ernzerhof (PBE) was employed to describe the electron exchange–correlation interactions [33,34]. The cut-off of the plane-wave kinetic energy and the convergence of total energy were set to be 400 eV and 10^−5^ eV. All studied layers were modeled in a rectangular supercell and located in the *x*-*y* plane. We adopted 17 × 11 × 1 *k*-point meshes with a Gamma centered grid to approximate Brillouin zone integrations. Because of the application of periodic boundary conditions, a vacuum region of over 10 Å was applied along the *z*-axis to eliminate the interactions between neighbor layers. Structural relaxations were performed by computing the Hellmann–Feynman forces using a conjugate gradient algorithm within a force convergence of 0.01 eV/Å [35].

As is well known, in a vertical stacking bilayer, long-range vdW interactions are crucial for maintaining the structure [36]. Herein, we used the DFT-D2 functional implemented in VASP to consider the vdW interaction [37]. For band structure calculations, the screened hybrid density functional of Heyd–Scuseria–Ernzerhof with 2006 parameterization (HSE06) was employed [38,39], which is an effective approach for predicting the band gaps of semiconductors [40,41]. In order to examine the stability of h-BP/borophosphene heterostructures induced by the vdW interaction, interlayer interaction energy was calculated according to the following definition:

$$E_{inter}=E_{H}-(E_{h\text{-}\mathrm{BP}}+E_{borophosphene}),$$

where $E_{H}$, $E_{h\text{-}\mathrm{BP}}$, and $E_{\mathrm{borophosphene}}$ are the total energies of the heterostructures, h-BP monolayer, and borophosphene, respectively. To further illustrate the vdW interaction, the difference in charge density was defined as in the following equation:

$$\Delta \rho =\rho_{H}-(\rho_{h\text{-}\mathrm{BP}}+\rho_{borophosphene}),$$

where $\rho_{H}$, $\rho_{h\text{-}\mathrm{BP}}$, and $\rho_{\mathrm{borophosphene}}$ are the charge densities of the heterostructures, h-BP monolayer, and borophosphene, respectively. By computing the complex dielectric function, optical absorption spectra were investigated. The absorption coefficient *I*(*ω*) was defined as follows [42]:

$$I(\omega )=\sqrt{2}\omega [\sqrt{\varepsilon_{1}(\omega {)}^{2}+\varepsilon_{2}(\omega {)}^{2}}-\varepsilon_{1}(\omega )]$$

where $\varepsilon_{1}(\omega )$ and $\varepsilon_{2}(\omega )$ are the real and imaginary parts of the dielectric function, and *ω* is a given frequency. The influence of excitons on optical properties was considered using the time-dependent DFT (TDDFT) method [43].

## 3. Results and Discussion

**Figure 1:**
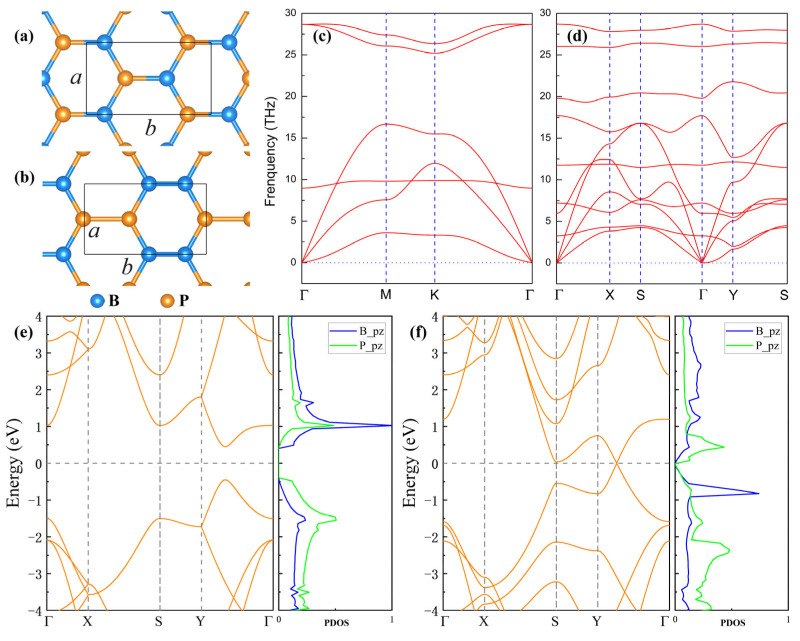
Geometrical structures (upper left panel), phonon spectra (upper right panel), and electronic band structures (lower panel) of h-BP monolayer (**a**,**c**,**e**) and borophosphene (**b**,**d**,**f**).

We first explored the geometrical structures and electronic properties of h-BP monolayer and borophosphene. Their optimized structures are shown in Figure 1a,b. Because of different atomic bonding interactions, the h-BP monolayer exhibited a hexagonal symmetry with the space group of *P-6m2* (187), while borophosphene displayed an orthorhombic lattice type with the space group of *Pmmm* (47). For ease of comparison, a rectangular lattice cell was adopted for both the borophosphene and h-BP monolayer. The optimized structural parameters and bond lengths are listed in Table 1. Despite the different geometrical symmetries, the structural parameters in both the borophosphene and h-BP monolayer were similar, as was the length of the B-P bond. The structural stability of borophosphene and the h-BP monolayer was analyzed according to the phonon modes shown in Figure 1c,d. It was found that no imaginary modes of lattice vibrations appeared in the whole Brillouin zone, indicating that both the borophosphene and BP monolayer were stable. Note that, in our previous study [21], borophosphene was demonstrated to be dynamically, thermally, and mechanically stable.

Although both the borophosphene and h-BP monolayer exhibit the structural characteristics of a graphene-like hexagonal honeycomb, their electronic band structures are quite different. As shown in Figure 1e,f, the h-BP monolayer is a direct band gap semiconductor with a band gap of 0.903 eV. By using a high-precision HSE06 method, a larger band gap of 1.371 eV is obtained, which aligns well with previous reports [44,45,46]. Of great interest is that the borophosphene is semi-metallic, exhibiting a Dirac Cone between the *Γ* and *X* points. Further analysis from the partial density of states indicates that the Dirac Cone is mainly induced by the π and π* interactions of *p*_z_ orbitals from B-B and B-P bonds. The difference in electronic properties between h-BP and borophosphene mainly arises from their different chemical bonds and structural symmetries. Note that bulk zinc-blende BP is an indirect band gap semiconductor with a band gap of 2.02 eV [47,48]. Thence, in the BP system, the reduction in dimensionality from 3D to 2D not only decreases the band gap but also can convert into a direct band gap semiconductor.

**Figure 2:**
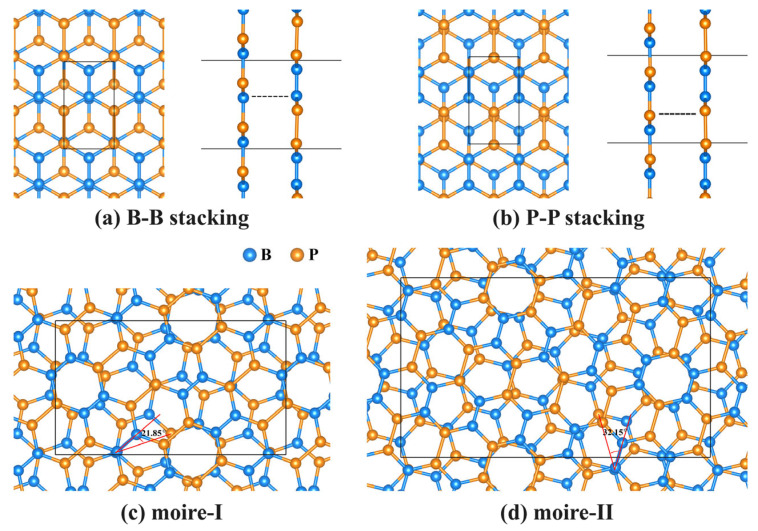
Optimized geometrical structures of h-BP/borophosphene heterostructures with different stacking orders.

**Figure 3:**
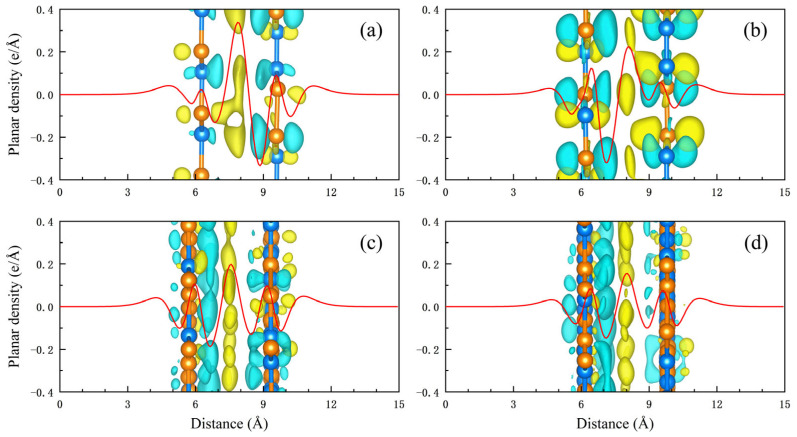
Average planar charge density for h-BP/borophosphene heterostructured bilayers: (**a**) B-B stacking, (**b**) P-P stacking, (**c**) moire-I, and (**d**) moire-II. Insets are differences in spatial charge density. The isosurface value is 0.0002 *e*/Å^3^. Yellow and light blue symbolize the gain and loss of electrons.

Because of their similar structural parameters, h-BP/borophosphene heterostructured bilayers can be constructed with negligible lattice mismatches of 0.25% and 0.04% along zigzag and armchair directions. Four types of vdW heterostructures with different stacking orders were considered and are shown in Figure 2. Both B-B and P-P stacking heterostructures exhibit an AB stacking sequence analogous to bilayer graphene. Specifically, for the B-B stacking bilayer, one B atom in the h-BP monolayer is vertically aligned with one B atom of borophosphene. In the P-P stacking bilayer, one P atom in the h-BP monolayer is directly superimposed over one P atom of borophosphene. In comparison with the individual monolayer, both the P-P and B-B stacking bilayers exhibit slight lattice contractions in both the armchair and zigzag directions. The interlayer distance of the B-B stacking bilayer is 3.340 Å, a little smaller than the 3.515 Å of the P-P stacking bilayer, indicating a stronger interlayer interaction of the B-B stacking heterostructure. Within the B-B stacking configuration, moire-I and moire-II heterostructures can be engineered through rotating borophosphene by 21.85° and 32.15°, exhibiting interlayer distances of 3.582 Å and 3.593 Å, respectively. Notably, these distance values are intermediate compared to reported interlayer spacings in related systems: 3.3 Å in a borophene/graphene heterostructure [15], 2.75 Å in blue phosphorene/borophene [49], 3.612 Å in phosphorene/graphene [17], and 3.40 Å in few-layer graphene [50]. The moderate interlayer distances in h-BP/borophosphene heterostructures suggest enhanced interlayer interactions between the h-BP monolayer and borophosphene. The interlayer interaction energies for B-B stacking, P-P stacking, moire-I, and moire-II heterostructures are −0.017, −0.014, −0.013, and −0.013 eV/Å^2^, respectively, which are comparable to −0.024 eV/Å^2^ of graphene on Cu (111) surface and −0.012 eV/Å^2^ of graphite on Cu (111) surface [51,52]. As illustrated in Figure 3, the average planar density indicates charge transfer and redistribution induced by vdW interactions for all heterostructured bilayers. A pronounced peak of charge accumulation is observed in the interlayer space, with the peak value diminishing as the interlayer spacing increases. The highest peak occurs in the B-B stacking bilayer, whereas the lowest is found in the moire-II bilayer, which reflects the varying strength of interlayer interactions. It is important to note that the charge transfer and redistribution are not determined by atomic electronegativity but are highly dependent on the stacking orders of h-BP/borophosphene heterostructured bilayers.

**Figure 4:**
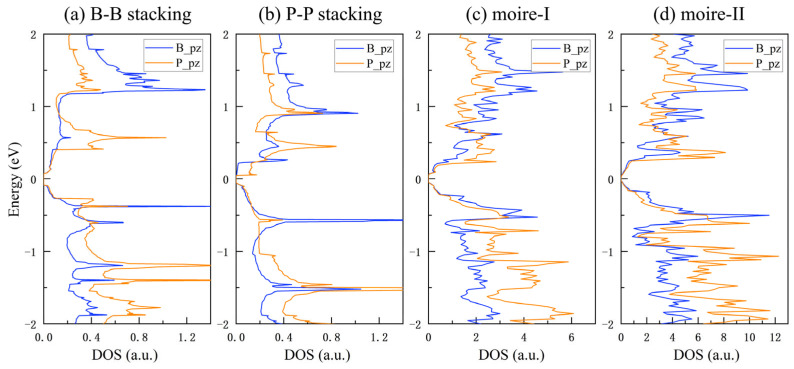
Partial density of states (DOS) for h-BP/borophosphene heterostructured bilayers. Since other orbitals are located at deeper energy levels, only the *p*_z_ orbital is presented.

Figure 4 displays the PDOS of the h-BP/borophosphene van der Waals heterostructures. Notably, all these heterostructures exhibit semiconducting properties characterized by a small band gap. As detailed in Table 1, the band gaps for the B-B stacking, P-P stacking, moire-I, and moire-II bilayers are 0.157, 0.101, 0.095, and 0.046 eV, respectively, and are closely related to the variations in interlayer spacing. Utilizing the HSE06 method, the predicted band gaps for the B-B and P-P stacking bilayers are 0.186 and 0.170 eV, indicating that these heterostructured bilayers possess the functionality of narrow band gap semiconductors. Further insights into the semiconducting characteristics of these bilayers can be derived from the PDOS and the charge density difference. In the B-B stacking bilayer, the regular atomic arrangement enhances interlayer dipole interactions, facilitating the transfer of the *p*_z_ orbital and resulting in a pronounced accumulation peak. This significantly modifies the π and π* interactions of the Dirac cone in borophosphene, opening a relatively large band gap of 0.157 eV. Conversely, the unique superlattice structure in the moire-II bilayer disperses the *p*_z_ orbital distribution through periodic potential modulation, thereby reducing the accumulation intensity and resulting in a very small band gap of 0.045 eV. Thence, it can be seen that the stacking orders play a crucial role in modulating interlayer interactions and tuning the band gap in h-BP/borophosphene vdW heterostructured bilayers.

**Figure 5:**
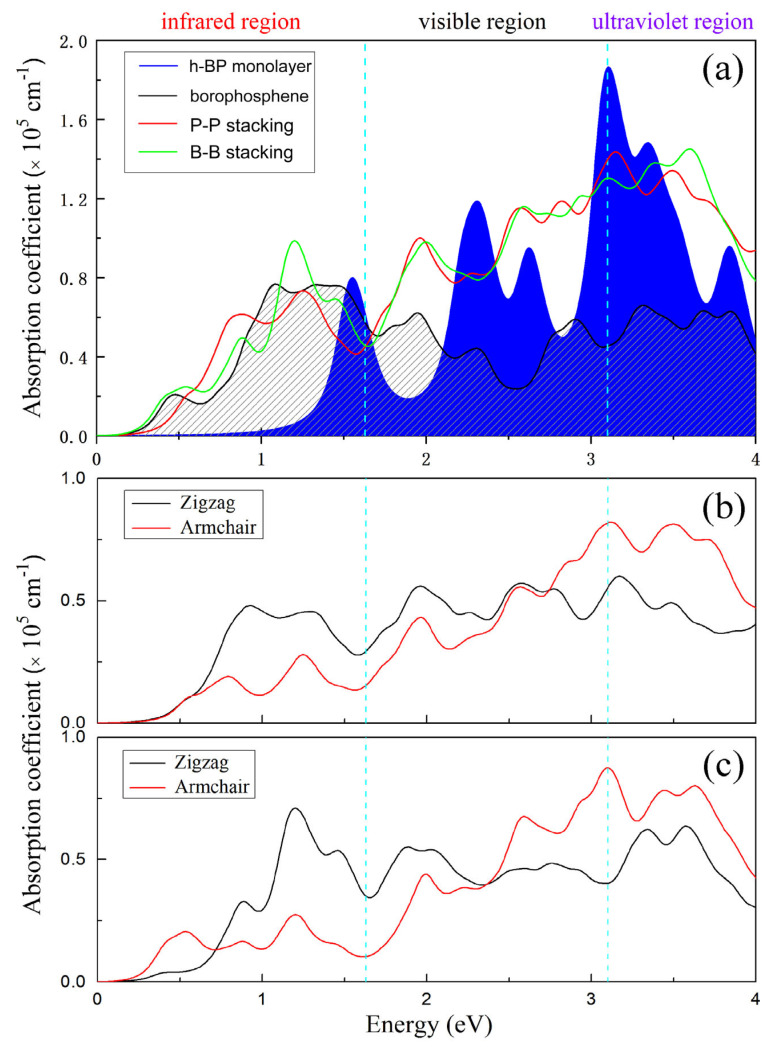
Optical absorption spectra (**a**) for h-BP monolayer, borophosphene, and h-BP/borophosphene heterostructures with P-P and B-B stacking orders. The optical absorption spectra along zigzag and armchair directions for (**b**) P-P and (**c**) B-B stacking heterostructures.

The optical absorption spectra of h-BP/borophosphene heterostructures are displayed in Figure 5a. For comparison, the optical absorption spectra of the h-BP monolayer and borophosphene are also shown. One can see that both the P-P and B-B stacking heterostructures exhibit similar absorption spectra in the visible and ultraviolet regions. A slight difference in spectra is observed in the infrared region. It should be pointed out that in the visible region, the optical absorption performances of P-P and B-B stacking heterostructures have been improved compared to the individual h-BP monolayer and borophosphene. The enhancement of optical absorption spectra is not merely a simple combination of the individual monolayers but is significantly influenced by the vdW interaction. A previous study has demonstrated that the absorption intensity of the constructed heterostructure surpasses that of an individual Pt_2_HgSe_3_ monolayer and graphene [53], indicating a distinct dielectric function and an improved absorption intensity. Figure 5b,c display the optical absorption spectra of the P-P and B-B stacking heterostructures for light polarization along the armchair and zigzag directions. Both heterostructures exhibit optical anisotropy, independent of stacking order. Along the armchair direction, similar absorption spectra are represented in the whole region. However, due to the different stacking orders and the vdW interaction, the optical absorption spectra are different along the zigzag direction, particularly in the infrared region.

## 4. Conclusions

In summary, we have systematically investigated the structural stability and the electronic and optical properties of h-BP/borophosphene vdW heterostructures with various stacking orders using a first-principles study. The results indicate that (i) the h-BP monolayer and borophosphene can form stable vdW heterostructures with suitable interlayer binding energies and negligible lattice mismatches (no larger than 0.25%); (ii) all vdW heterostructures exhibit semiconducting characteristics with small band gaps, ranging from 0.046 to 0.157 eV, as predicted using the PBE functional; (iii) interlayer stacking orders significantly influence the band gaps. Specifically, B-B and P-P stacking heterostructures, characterized by regular atomic arrangements and strong interlayer interactions, exhibit larger band gaps. In contrast, moire-I and moire-II heterostructures, which possess irregular atomic arrangements and weaker interlayer interactions, display smaller band gaps; (iv) compared to the individual h-BP monolayer and borophosphene, the optical absorption properties of h-BP/borophosphene heterostructures are enhanced due to vdW interactions.

## Figures and Tables

**Table 1:** Properties of h-BP monolayer, borophosphene, and h-BP/borophosphene heterostructures with different stacking orders: structural parameter (Å), bond length (Å), interlayer interaction energy *E_inter_* (eV/Å^2^), and band gap *E*_g_ (eV) predicted by PBE and HSE06.

Structures	Structural Parameters			*E_inter_*	*E*_g_	
	*a*	*b*	*d*		PBE	HSE06
h-BP monolayer	3.213	5.564	-	-	0.903	1.371
borophosphene	3.221	5.566	-	-	-	-
B-B stacking	3.210	5.556	3.340	−0.017	0.157	0.186
P-P stacking	3.209	5.555	3.515	−0.014	0.101	0.170
moire-I	14.706	8.492	3.582	−0.013	0.095	-
moire-II	20.049	11.575	3.593	−0.013	0.046	-

## Data Availability

Data are contained within the article.
